# Efficacy of acupuncture at three nasal acupoints plus acupoint application for perennial allergic rhinitis: A multicenter, randomized controlled trial protocol

**DOI:** 10.1186/s13063-019-4039-3

**Published:** 2020-01-28

**Authors:** Yin Shou, Li Hu, Cuihong Zhang, Shifen Xu, Qi Jin, Li Huang, Bingrong Li, Long Yuan, Siwei Xu, Kaiyong Zhang, Huiru Jiang, Bimeng Zhang

**Affiliations:** 1 Department of Acupuncture and Moxibustion, Shanghai General Hospital, Shanghai Jiaotong University School of Medicine, Shanghai, China; 2 Acumox and Tuina Research Section, College of Acumox and Tuina, Shanghai University of Traditional Chinese Medicine, Shanghai, China; 3 Shanghai Research Institute of Acupuncture and Meridians, Shanghai, China; 4 Acupuncture Department, Shanghai Municipal Hospital of Traditional Chinese Medicine Affiliated to Shanghai University of TCM, Shanghai, China

**Keywords:** Acupuncture at three nasal acupoints, Acupoint application, Perennial allergic rhinitis, Randomized controlled trial, Visual Analog Scale

## Abstract

**Background:**

Many studies have shown the potential therapeutic effect of acupuncture on allergic rhinitis. Most of these studies were limited by low-quality evidence. Preliminary experiments showed that the use of acupuncture at three nasal acupoints plus acupoint application (AAP) achieves a more persistent effect in the treatment of perennial allergic rhinitis than acupuncture alone. In this study, a multicenter, single-blind, randomized controlled trial will be performed, in which acupuncture at nonmeridian acupoints and sham AAP will be used as the control group to evaluate the effect of AAP through long-term observation.

**Methods:**

The trial is designed on the basis of the Consolidated Standards of Reporting Trials 2010 guidelines and Standards for Reporting Interventions in Controlled Trials of Acupuncture. A total of 120 participants with perennial allergic rhinitis will be randomly assigned to a treatment or control group. A specially appointed investigator will be in charge of randomization. The participants in the treatment group will be treated with acupuncture at EX-HN3, LI20, and EX-HN8 thrice per week for a total of 12 sessions. In addition, they will undergo AAP at DU14, BL13, EX-BI, and RN22. The participants in the control group will be treated with sham AAP. The primary outcome will be the change in the Total Nasal Symptom Score from baseline to the completion of 4-week treatment. Secondary outcomes include changes in visual analog scale and total non-nasal symptom scores from baseline to the second and fourth weeks of treatment, as well as 1, 3, and 6 months after the completion of treatment. Peripheral blood IL-4, IL-5, IL-6, IL-8, and IL-10 levels will be measured, and any side effects related to treatment will be observed and recorded.

**Discussion:**

It is expected that this randomized clinical trial will provide evidence to determine the effects of AAP compared with acupuncture at nonmeridian acupoints and sham AAP, particularly the long-term effect. These findings will help improve the clinical application of this technique.

**Trial registration:**

Acupuncture-Moxibustion Clinical Trial Registry AMCTR-ICR-18000179. Registered on 12 April 2018.

## Background

Allergic rhinitis (AR) is a symptomatic nasal disorder resulting from an IgE-mediated immunological reaction to allergen exposure [1]. The clinical manifestation of AR includes rhinorrhea, nasal pruritus, nasal obstruction, and excessive sneezing. Other associated symptoms include postnasal drip, ocular pain, headache, and insomnia. AR has adverse impacts on sleep, cognitive functioning, mood, and other associated comorbidities such as asthma and sinusitis, ultimately affecting quality of life, as well as work and school performance [2]. According to epidemiological research findings, AR affects up to 40% of the global population [3, 4] and 11.1–17.6% of the Chinese population [5], affecting the quality of life of patients [6] and causing a significant socioeconomic burden [7]. Proper classification facilitates selecting the most appropriate treatment strategies for AR patients [1, 8, 9]. AR may be classified by (i) temporal patterns of exposure to trigger allergens such as seasonal (e.g., pollens), perennial/year-round (e.g., dust mites), and episodic allergens (e.g., irregular environmental exposures such as visiting a home with pets); (ii) frequency of symptoms; and (iii) severity of symptoms. AR severity can be classified from mild (when symptoms are present but do not interfere with quality of life) to more severe (when symptoms interfere with quality of life) [10]. The pathogenesis of AR can be divided into four phases: sensitization, subsequent reaction to allergen, late-phase activation, and systemic activation [9].

Controlling symptoms has traditionally been the main goal of AR management due to elusiveness of definitive cure [11]. The current mainstream AR managements include avoidance of allergens and other trigger factors, pharmacotherapy, immunotherapy, and desensitization. Despite the methodological limitations in the few economic evaluations that have been conducted, evidence appears to support the cost-effectiveness of subcutaneous immunotherapy rather than of pharmacotherapy [12]. However, these treatments do not always provide complete symptom relief and are associated with undesirable side effects. Pharmacotherapy provides only rapid symptom relief from perennial AR (PAR) and becomes less effective when recurrently used due to the development of drug tolerance [13, 14].

Complementary and alternative medicine (CAM) therapies, including Chinese herbal medicine, Ayurvedic medicine, other single and multiple herbal preparations, acupuncture, homeopathy, and several other modalities, continue to gain popularity in the treatment of AR. Reportedly, > 42% of Americans have used CAM for AR, and the popularity of CAM therapies for allergic diseases is even greater in some European countries [15, 16]. A hospital survey conducted in Japan found that 19.2% of participants had, at some point, undergone CAM therapy and that approximately 36.2% of these believed that the therapy was effective. Respondents cited safety, convenience, and low price as the main reasons for using CAM [17]. In a 2018 survey of allergists, 81% responded that they had patients who use CAM therapies and that patients use CAM therapies more often than vitamin supplements [18]. The chronic nature of allergic diseases and the paucity of preventive or curative treatments also stimulate interest in CAM therapies [19].

Given the popularity of CAM treatments, high-quality data about these therapies are needed to establish professional practice guidelines. In the United States, the National Center for Complementary and Integrative Health has been tasked with evaluating the mechanisms, efficacy, and safety of botanical medicines through basic science studies, clinical research, and the establishment of dedicated botanical research centers [20]. The design of randomized, placebo-controlled studies in CAM is complicated by difficulties in blinding and the establishment of appropriate placebos, particularly in trials of acupuncture.

Acupuncture is a component of traditional Chinese medicine (TCM) that was originally believed to work on the principle of the redistribution of qi, the life energy. In TCM, disease is understood to originate from an imbalance or poor flow of qi. Acupuncture has long been used in TCM to treat AR, and studies of this treatment have shown mixed results, with the most rigorous studies showing only modest clinical benefits [21–24]. The 2015 revision of the American Academy of Otolaryngology-Head and Neck Surgery clinical practice guidelines for AR cites a low level of confidence in the evidence to support acupuncture as a treatment option. Acupuncture may be a reasonable option for patients with relatively mild symptoms who wish to minimize medication use and who find its cost acceptable [1]. These results have helped practitioners to improve their use of acupuncture to treat AR. However, high-quality scientific evidence is needed to instruct the clinical application of acupuncture.

Preliminary experiments have found that the use of acupuncture at three nasal acupoints plus acupoint application (AAP) achieve a more persistent effect in the treatment of AR than simple acupuncture alone. Based on this preliminary research, a multicenter, randomized controlled trial (RCT) has been designed in which acupuncture at nonmeridian acupoints and sham AAP will be used with a control group to evaluate the effect of AAP through long-term observation. The purpose of this trial is to answer three questions: (1) Does AAP benefit PAR? (2) How long do the effects last? and (3) Does AAP work by intervening peripheral blood IL-4, IL-5, IL-6, IL-8, and IL-10 levels? Similar research techniques have been applied in previous studies on PAR such as the Total Nasal Symptom Score (TNSS) [25], visual analog scale (VAS) [26], and Total Non-Nasal Symptom Score (TNNSS) [22]. This trial has adopted TNSS, VAS, and TNNSS due to their wide applicability. The trial hypothesis is that AAP will achieve equal or better long-term symptom relief in PAR or severe AR and reduce its recurrence compared to acupuncture alone.

The trial has been registered at acmctr.org AMCTR-ICR-18000179.

## Methods/design

### Design

**Fig. 1:**
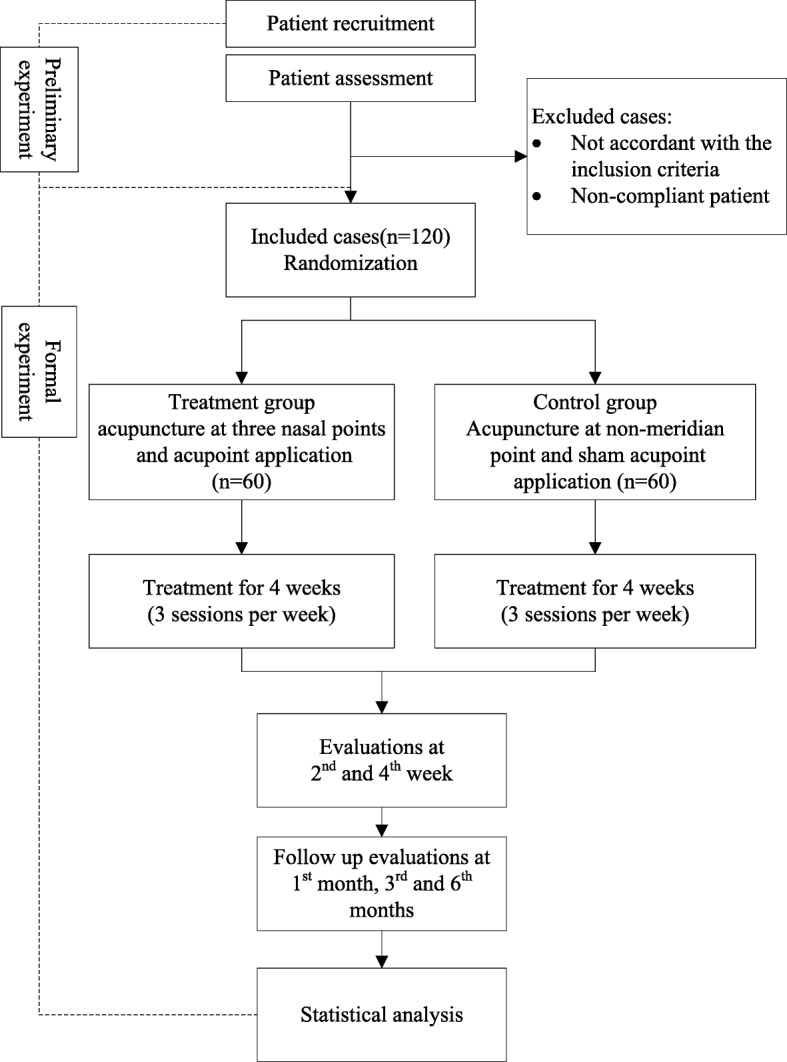
CONSORT flow diagram

**Fig. 2:**
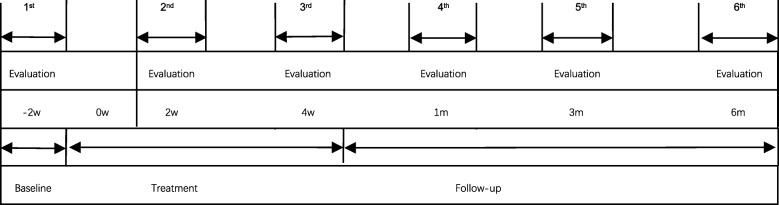
Total trial period and evaluation time points

The study is designed as a multicenter, parallel-group, randomized, and single-blind trial to compare AAP with a sham acupuncture for PAR treatment. The trial was designed according to the Standard Protocol Items: Recommendations for Interventional Trials (SPIRIT) statement (Fig. 2) [27], and conforms to the Consolidated Standards of Reporting Trials (CONSORT 2010) guidelines (Fig. 1) and Standards for Reporting Interventions in Controlled Trials of Acupuncture (STRICTA) [28, 29].

To achieve the targeted enrollment of participants, clinical recruitment flyers will be placed at three hospitals. Clinical recruitment staffs are responsible for enrolling 120 participants with moderate-to-severe PAR. Participants will be screened based on inclusion and exclusion criteria. All participants will be briefed on the purpose, procedures, treatments, and possible risks of the trial; and will be clearly informed of their rights to discontinue participation at any point. Screening will take approximately 1 week. During this period, participants will be asked to complete questionnaires related to their symptoms. Once participants have signed consent forms, they will be randomized to receive 12 sessions of AAP or sham acupuncture and AAP for 4 weeks.

The trial design flowchart is presented in Fig. 1, and the trial design schedule is shown in Table 1.

**Table 1:** Trial design schedule

Period	Screening	Baseline	Treatment(W1–4)		Follow-up(W5-M6)	
Time	W-1	W0	W2	W4	M3	M6
Eligibility	X					
Demography and medical history	X					
Physical examination	X					
Informed consent	X					
TNSS	X	X	X	X	X	X
VAS	X	X	X	X	X	X
TNNSS	X	X	X	X	X	X
Adverse event			X	X		
Medication use	X	X	X	X	X	X
Compliance			X	X	X	X

*TNSS* Total Nasal Symptom Score, *VAS* visual analog scale, and *TNNSS* Total Non-Nasal Symptom Score

### Trial location

Participants will be recruited from three hospitals:

1. Shanghai General Hospital, Shanghai Jiao Tong University School of Medicine
2. Shanghai Municipal Hospital of Traditional Chinese Medicine Affiliated to Shanghai University of Traditional Chinese Medicine
3. Shanghai Research Institute of Acupuncture and Meridians

All three research centers are located in Shanghai, China.

### Trial participants

The trial will recruit inpatients from the three trial locations admitted from January 2018 to June 2020. To ensure that results are accurate, the trial will include strict inclusion, exclusion, and elimination criteria, as outlined below.

### Inclusion criteria

Participants are considered eligible if they meet all of the following criteria: (1) meet the diagnostic criteria for moderate-to-severe PAR, (2) meet the diagnostic criteria for PAR lung Qi deficiency and cold syndrome, (3) any gender, aged 18–60 years old, (4) PAR that could be distinguished from cold or tetanus-related motor rhinitis, and (5) agree to participate in the trial with providence of informed consent.

### Exclusion criteria

Participants will be excluded if they meet any of the following criteria: (1) does not meet the diagnostic criteria for PAR; (2) presence of rhinosinusitis, sinusitis, and nasal septal deviation as comorbidities; (3) presence of severe respiratory illness, circulatory disease, digestive disease, urinary system disease, hematologic disease, nervous system disease, endocrine disease, mental disorder, or malignant tumor as comorbidities; (4) underwent antibiotic treatments for upper respiratory tract infection or paranasal sinusitis in the 2 weeks prior to the start of the trial; (5) pregnant or lactating women; (6) presence of skin lesions or scars on the AAP acupoint; (7) particularly sensitivity to drugs or dressings; (8) pretreatment TNSS of < 4; and (9) presence of alcoholism, which cannot be terminated during the experiment.

### Elimination criteria

Trial participants will be allowed to or will be asked to leave the trial if (1) a participant has been included in the trial but cannot, at any stage in the trial, be subjected to the assigned treatment for various reasons, (2) severe adverse events occur that necessitate the withdrawal of a participant from the trial, (3) a participant does not fully participate in treatment or follow-up, (4) a participant does not match the inclusion criteria but has been accidentally included, and (5) a participant does not comply with the treatment or fails to provide information that may be important to the evaluation.

### Ethics

This trial has been approved by the Ethics Committee of the Institute of Shanghai General Hospital, Shanghai Jiao Tong University School of Medicine ([2017]31), Shanghai Municipal Hospital of Traditional Chinese Medicine (2017SHL-KY-06), and Shanghai Research Institute of Acupuncture and Meridians (2017–037-01). After obtaining ethics committee approval, the trial was registered on an authoritative registration platform for clinical trials (Acupuncture and Moxibustion Clinical Trial Registry, AMCTR-ICR-18000179). The informed consent form was developed in accordance with the Declaration of Helsinki.

Research assistants will thoroughly explain the purpose, procedure, treatment, and possible risks of the trial to participants, and they will clearly inform participants of their right to discontinue the trial at any point. Participants will be required to sign the informed consent form before the start of the trial. Research assistants will be in charge of the storage of all informed consent forms.

This is the protocol of the second version (v2.0 dated June 19, 2017). Based on the first version, the exclusion criteria have been slightly modified. All protocol versions have been submitted to the ethics committees of the three hospitals. Approvals from the research ethics committee review boards have been retained by the ethics committees of the three hospitals.

### Randomization

Participants will be randomly assigned at a ratio of 1:1 to a treatment or control group. The randomization sequence was generated using block randomization with a table of randomization. The table contains, in a random order, all possible combinations of a small series of figures, and it assumes an equal probability of a participant being randomly assigned to the treatment or control group. The order of interventions assigned to each block is randomized. The process is repeated for consecutive blocks until all participants are randomized. While receiving the first treatment, participants will be given sequential treatment cards from independent researchers to ensure adequate concealment.

### Blinding

This will be a single-blind trial. All participants will be treated separately to prevent communication. Except acupuncturists, all relevant parties will be blinded to the intervention groups. Treatments will be administered by two acupuncturists using treatment patches prepared by operational assistants. Due to the nature of AAP, it is difficult to fully ensure blinding among participants allocated to either group. Participants will be required to wait for 120 min in a room, after which their treatment patches will be removed by research nurses. In addition, acupuncturists, operational assistants, and research nurses are instructed to not communicate with participants on any information that might alert them to which group they have been allocated. Participants will be informed that they have an equal chance of allocation to the treatment or control group before research participation. The participants will be blinded to the group they belong to because needle penetration is achieved in all cases. In addition, outcome evaluators and statistical analysts will be blinded to the groupings and will not be involved in any part of the treatments during trial to ensure no statistical bias in the results.

### Intervention

#### Treatment group

All licensed acupuncturists have completed at least 5 years of undergraduate education and are registered TCM practitioners. All research assistants and licensed acupuncturists involved in the trial will receive a 2-day training session prior to the start of the trial. Both treatments will comprise 12 sessions, each lasting for 30 min, administered regularly over a 4-week period. The licensed acupuncturists will provide AAP thrice a week for 4 weeks.

The treatment group will receive AAP. Disposable and sterile 0.25 × 40 mm acupuncture needles (Suzhou Tianxie Acupuncture Instruments Co., Ltd., Suzhou, China) will be used. Acupoints include Yintang (EX-HN3), Yingxiang (LI20), and Shangyingxiang (EX-HN8) as well as Feishu (BL13), Dazhu (BL11), Fengmen (BL12), Taiyuan (LU9), and Zusanli (ST36), which will be located according to World Health Organization (WHO) International Standard Acupuncture Points. Participants will be positioned in the seated position during treatment sessions. After routine skin sterilization, needles will be inserted using the neutral reinforcing–reduced manipulation technique. Each needle will be rotated until the participant experiences the qi sensations of soreness, heaviness, and distension. The complete AAP formula used in this trial is not publicly available, but the primary herbal ingredients include *Rhizoma corydalis*, *Semen sinapis*, *Ephedra sinica*, *Cortex cinnamomi*, *Euphorbia kansui*, *Syzygium aromaticum*, and *Asarum sieboldii* Miq. These herbs are processed into powder, proportionally mixed at a ratio of 2:2:1:1:1:1:1, and blended into fresh ginger juice to create an AAP ointment, which is then mechanically poured into tubes. The resulting AAP ointment is stored in a refrigerator at 4 °C. The AAP ointment and matching placebo are both manufactured by the Pharmaceutical Preparation Department of the First People’s Hospital Affiliated to Shanghai Jiaotong University, and both meet the regulatory guidance requirements issued by the China Food and Drug Administration. For each acupoint, approximately 3 g of ointment is squeezed by assistants onto a 6-cm-diameter circular fabric. For each participant, the following six acupoints will be used, which are located according to the WHO International Standard Acupuncture Points: Dazhui (GV14), Feishu (BL13), Dingchuan (EX-B), and Tiantu (CV22). The treatment procedure has been standardized at each center by advance training (Table 2).

**Table 2:** Acupoints and needling procedure

Acupoints	Angle and direction	Depth (mm)
Yintang (EX-HN3)	Transversely, downward	13
Yingxiang (LI20)	Transversely, towards the root of nose	5
Shangyingxiang (EX-HN8)	Transversely, towards the nose	8
Feishu (BL13)	Obliquely	5
Dazhu (BL11)	Obliquely	5
Fengmen (BL12)	Obliquely	5
Taiyuan (LU9)	Straightly	5
Zusanli (ST36)	Straightly	13

#### Control group

The control group will receive shallow needling at sham Yintang (EX-HN3), sham Yingxiang (LI20), sham Shangyingxiang (EX-HN8), sham Feishu (BL13), sham Dazhu (BL11), and sham Taiyuan (LU9). Each of these acupoints is a nonacupoint located at a different physical location to the actual acupoints. The placebo ointment is composed of buckwheat powder and coke, resulting in an ointment appearing similar to the AAP ointment.

#### Concomitant care and intervention

In both groups, participants with severe symptoms will be allowed to use rescue medicine with documentation of the medication. The type of medicine, dosage, and usage will be recorded on diary cards for analysis. For more complicated chronic diseases, participants must continue to take their routine medication and to receive the necessary therapies. In their case reports, the research staff will record the names of these diseases and the names of medications and therapies used.

#### Discontinuation of the intervention

The intervention should be terminated in the case of severe adverse events, withdrawal of participants, and unpermitted medication use.

### Outcome measures

#### Baseline information

Demographic information will be collected using a custom-made, standardized survey form that includes the following items: center location, name, age, sex, address, telephone number, and employment. A custom-made form will be used to collect medical information including diagnosis, nasal mucosal examination result, allergen examination result, typical symptoms, occurrence time of symptoms, accessory examination of nasal mucosa and nasal sinus, relevant diseases (allergic asthma and allergic conjunctivitis), and medication history.

#### Primary outcome measure

Change in TNSS will be measured for each participant by comparing the baseline score with the score at the end of the 4-week treatment. TNSS is determined by the severity of rhinorrhea, nasal pruritus, nasal obstruction, and sneezing.

#### Secondary outcome measures

Changes in TNSS will be measured by comparing the baseline score with the score at 2 weeks and 1, 3, and 6 months after treatment. VAS and TNNSS will be used to observe supplementary symptoms and severity of symptoms. These will also be evaluated at 2 and 4 weeks and 1, 3, and 6 months after treatment. Peripheral blood IL-4, IL-5, IL-6, IL-8, and IL-10 levels will be measured by Luminex to observe allergic reactions.

All outcome readings will be scored on quantitative scales and summarized as mean values and standard deviation.

#### Safety assessment

Adverse events (AEs) are defined as at least four participants with the same symptom, i.e., any undesirable experience occurring to participants during the trial period. This may or may not be associated with the intervention. Participants are instructed to report any AE to the research team at any time. All details of AEs including time of occurrence, description of symptoms, duration of symptoms, severity, management measures, and causality to the intervention will be recorded on case report forms (CRFs). Common AEs related to acupuncture include local skin pain, itching, ulcers, needle left in place, nausea during acupuncture, fainting during acupuncture, severe sharp pain, sharp pain lasting for > 30 min, hematoma around the site of needling, bleeding, numbness, infection around the site of needling, sleeplessness after acupuncture, and dizziness after acupuncture [30]. Common AEs related to AAP include local itching, redness, and blisters [31]. The causality between AEs and the intervention is assessed according to the WHO Uppsala Monitoring Center System for Standardized Case Causality Assessment [32]. If AEs occur, the research staff will select an appropriate treatment method until the condition has stabilized. After the participant’s condition returns to normal, the research staff will decide whether further observation is required. Severe AEs must be reported to the safety monitoring board within 24 h of their occurrence.

#### Follow-up

The 4-week treatment period will be followed by a 6-month follow-up period, during which the research staff will continue to follow participants’ progress via telephone communication and text message. The frequency of follow-up will be monthly, and records of symptom assessment and medication compliance or changes will be recorded. In the event of discontinuation or deviation from intervention protocols, the research staff will record the reasons and medication details and then exclude the participants and their latest outcome data including symptoms and frequency of AEs from the trial.

#### Recruitment timeline

For an overview of the recruitment timeline, interventions, and all time points of participant evaluation, see Fig. 2.

#### Data collection and management

The research staff will be responsible for the collection of baseline characteristic data and medical results during the screening period. For convenience, all participants’ scores, observation times, AE records, and safety assessments will be consolidated into a single CRF. CRFs must be filled out immediately and accurately after each piece of information is acquired. Participants are required to record in their daily diary any other medications they take during the trial period. Outcome evaluators will examine the outcomes at baseline, 2 weeks (within treatment), 4 weeks (end of treatment), 1 month (within follow-up), 3 months (within follow-up), and 6 months (end of follow-up). Data on nasal and non-nasal symptoms will be collected.

Data monitoring and management will be performed every 3 months by the Clinical Research Center of First People’s Hospital Affiliated to Shanghai Jiaotong University. The clinical research monitor will monitor medical practitioners to ensure all processes are correctly implemented. A data monitoring committee (DMC) has been established independent of the sponsor, and no conflict of interest exists. The DMC is responsible for monitoring trial progression and guaranteeing participant safety. Interim analyses and termination plans for the trial have not been specified, but if the DMC requests interim analyses, these will be supplied. Two assistants will enter all data into an electronic database by double data entry. The statistical manager will be responsible for source data organizing, coding, range checking for data values, and converting data to ensure data quality. The database will be locked after all data have been cleaned. If participants withdraw from the trial, the reasons should be detailed and the rate of withdrawal should be statistically analyzed.

#### Quality control

Before recruitment, the entire research staff involved in the trial including acupuncturists, operational assistants, and research nurses must receive training in advance. Training will include the method for correctly filling out CRFs, blood tests, details of AAP, use of scales, participants’ method of using the medication diary, and follow-up visit skills. Researchers will be examined after training to ensure strict adherence to the trial protocol and consistency of the trial administration process including acupuncture and AAP treatment and evaluation method. The entire research staff will be provided with a written protocol and standard operating procedure documents. All acupuncturists who apply the treatment must have acupuncture licenses from the Ministry of Health of People’s Republic of China and > 5-year clinical experience.

To improve the quality of trial reporting and conduct, we will develop a standard operating procedure manual according to the principles of the CONSORT Extension for Chinese Herbal Medicine Formulas [33] and will thoroughly train all investigators. Intervention details such as acupuncture rationale, needling details, treatment regimen, time selection, practitioner background and confidence, and adequacy of stimulation were extracted and evaluated by CZ and SX according to STRICTA [34].

To ensure data authenticity, a special research team from the Clinical Research Center of Shanghai General Hospital, Shanghai Jiao Tong University School of Medicine, independent of the investigators and sponsors, will externally monitor the trial at the three hospitals every 3 months. An advisory board will follow the trial and provide advice when necessary.

To improve intervention adherence, free treatment and blood tests will be provided to the treatment group for 4 weeks. To ensure that treatment and follow-up run on schedule, participants will be assured of and given monetary compensation at the end of the follow-up period.

### Statistical analysis

#### Sample size calculation

The trial will test two groups in parallel. Sample size calculation was performed using SAS 9.3 (SAS Institute Inc., Cary, NC, USA) at the Clinical Evaluation Center of First People’s Hospital Affiliated to Shanghai Jiaotong University. The mean change in TNSS before and after treatment was used as the indicator of efficacy evaluation in sample size calculation. Other research has shown a mean TNSS change of 2.53 ± 4.74 after acupuncture treatment [22] and 2.75 ± 1.06 after AAP [35]. Based on these findings, the appropriate trial sample size with power of 80%, alpha value of 0.05, and acceptable delta value of 0.2 was calculated. The results show that a clinically important difference can be detected by a sample size with at least 49 in each group. Accordingly, this number was increased to 60 per group (a total of 120) to allow for a predicted 20% dropout rate.

#### Analysis procedures

Statistical analysis will be performed using SPSS 16.0 (SPSS Inc., Chicago, IL, USA) at the Clinical Evaluation Center of Shanghai General Hospital, Shanghai Jiao Tong University School of Medicine.

#### Sample distribution

The size and dropout rate for each dataset will be described. Clarified reasons for any participant’s withdrawal from the trial will be provided.

#### Baseline information

Baseline adjusted analyses will be provided for center and severity variables, and the baseline value of the corresponding outcomes will be assessed. Descriptive statistics will be used to compare baseline measures with participant characteristics. If an imbalance occurs in baseline characteristics between the two groups, analysis of covariance will be applied.

#### Efficacy analysis

Efficacy data analyses will be conducted on an intention-to-treat population. All participants initially included in any group will be considered in statistical analysis. Analysis of efficacy will be performed per-protocol and will include all participants who complete the entire research. Descriptive statistics will be used to compare serum indicators between the two groups. Regarding the primary and secondary outcome measures, a two-sample *t* test or Wilcoxon rank-sum test will be used to compare differences between both groups from baseline to the end of treatment (*p* < 0.05 will be considered statistically significant). The mean and standard deviation values of these parameters will be reported. Regarding repeated measures data, repeated measures analysis of variance will be performed after meeting spherical symmetry requirements. SPSS 16.0 will be used for all statistical calculations.

#### Safety analysis

According to the definition of AEs, AEs will be recorded, along with their severity level, causes, and explanations. The number of AEs and rate of AEs will be described statistically. If AEs need to be compared between groups, χ^2^ test or Fisher’s exact test will be used.

#### Missing data analysis

All data used in the main statistical analysis should be collected by the fourth week of the treatment and by the 6-month follow-up. To avoid missing data, participants who complete the trial and provide completed data will be financially compensated. The investigators have a wealth of experience from previous trials in managing participants and collecting data. Participant contact information will be recorded and researchers will keep in touch with them through various means of communication during the treatment and follow-up periods.

If data are not obtained, the time and reason for missing data will be recorded and the assumed missing data mechanism will be analyzed. For these missing data, a multiple imputation adjustment approach will be used. After the main analysis, sensitivity analysis will be performed for the various datasets to enable the assessment of the impact of missing data on the results.

A detailed statistical analysis plan will be written by an independent statistician.

#### Publication and dissemination

Following the completion of data analysis, Chinese and English dissemination is planned. Regardless of the findings, the trial results with be disseminated via conferences or publications.

The entire research staff who participated in the organization, implementation, data management, and statistical analysis will be affirmed in the authorship, and there is no intention to use professional writing services.

There is no plan to permit public access to the full protocol, participant dataset, or statistical code. However, if necessary, individuals can gain access to the full protocol through the Ethics Committee of the Institute of Shanghai General Hospital Affiliated to Shanghai Jiaotong University.

This protocol was written following the SPIRIT checklist (see Additional file 1). The future report will follow the CONSORT guidelines [29], Revised STRICTA guidelines [28], and extension of CONSORT for reporting single-blind randomized trials.

## Discussion

Acupuncture is an important component of TCM. It is technically simple to perform and easy to teach. Research has indicated that acupuncture alleviates symptoms of PAR and improves quality of life [36]. However, few high-quality studies support the technique in this regard [37]. Standardized acupuncture treatment protocol and scoring system are currently not available; therefore, it is difficult to draw conclusive evidence through systemic review and meta-analysis [36]. Procedure and therapeutic effects of acupuncture are based on thousands of years of empirical practice. With the worldwide applications of acupuncture, advanced molecular biology methods have been used to explore the mechanism. However, because TCM is based on a different philosophy to biomedicine, incorporating acupuncture into treatment regimens remains difficult. Nevertheless, recent advances in neuroendocrinology and immunology have allowed a better understanding of acupuncture. The results of this trial will help determine whether this treatment should be more widely applied in clinical practice.

In the preliminary experiment, a pilot RCT was conducted using a small sample with the aim of testing the feasibility of the trial. Participants were divided into three groups of 20. The first group underwent AAP, the second underwent acupuncture only, and the third underwent loratadine therapy. The results showed that AAP achieved a more persistent effect than acupuncture or loratadine therapy alone. This trial intends to use this multicenter RCT to provide reliable evidence for the short-term and long-term efficacy of AAP. This will be compared with a control group of acupuncture at nonmeridian acupoints and sham AAP in the treatment of AR. The trial will evaluate differences in the short-term and long-term effects of AAP and whether this treatment can affect peripheral blood IL-4, IL-5, IL-6, IL-8, and IL-10 levels. TNSS determined at the end of the 4-week treatment will be taken as the primary outcome of the trial. This will enable further integration of acupuncture into the current scientific practice. Therefore, the trial presents a promising new approach to the fusion of acupuncture and biomedicine using AAP as an example.

### Trial status

The recruitment of participants for this trial has begun. The trial is designed to be completed by June 30, 2020.

## Supplementary information

**Additional file 1.** SPIRIT 2013 checklist.

## Data Availability

Data sharing will be publicly open within 12 months after the completion of the trail.

## Abbreviations

AAP: Acupuncture at three nasal acupoints plus acupoint application
CAM: Complementary and alternative medicine
PAR: Perennial allergic rhinitis
RCT: Randomized controlled trial
TCM: Traditional Chinese medicine
TNNSS: Total Non-Nasal Symptom Score
TNSS: Total Nasal Symptom Score
VAS: Visual Analog Scale
