# Supplementary material for: Efficacy of acupuncture at three nasal acupoints plus acupoint application for perennial allergic rhinitis: A multicenter, randomized controlled trial protocol
Source: Trials. 2020 Jan 28;21:110. doi: 10.1186/s13063-019-4039-3 (PMC6986062; doi:10.1186/s13063-019-4039-3)
Supplement: Supplementary file 1 — Additional file 1. SPIRIT 2013 checklist. [file 13063_2019_4039_MOESM1_ESM.docx]

**Additional File 1**

SPIRIT 2013 Checklist: Recommended items to address in a clinical trial protocol, and related documents*

| **Section/item** | **Item No** | **Description** |
| --- | --- | --- |
| Administrative information | | |
| Title | 1 | Efficacy of using acupuncture at three nasal points plus acupoint application for perennial allergic rhinitis: study protocol for a multicenter randomized controlled trial |
| Trial registration | 2a | Acupuncture Clinical Trail Registry, AMCTR-ICR-18000179 |
|  | 2b | All items from the World Health Organization Trial Registration Data Set. |
| Protocol version | 3 | 2018/6/17 14:14:21, 1008001 Prospective registration |
| Funding | 4 | This work is supported by grants from the Science and Technology Commission of Shanghai Municipality (No. 17401932200), Shanghai Municipal Health Bureau (No. ZYBZ-2017026, ZY2018-2020-FWTX-4029, ZY2018-2020-FWTX-4029), Science and Technology Commission of District Songjiang Shanghai (No. 2017-33), and the National Natural Science Foundation of China (81403470,81600611).The role of the funding body is in the design of the study, collection, analysis, interpretation of data, the writing of the manuscript. |
| Roles and responsibilities | 5a | Yin Shou1,2†, Li Hu2†,Cuihong Zhang3, Shifen Xu4,Qi Jin4, Li Huang4, Bingrong Li1, Long Yuan1, Siwei Xu1, Kaiyong Zhang1, Huiru Jiang1, Bimeng Zhang1 *   1. Department of Acupuncture-Moxibustion, Shanghai General Hospital, Shanghai Jiaotong University, Shanghai , China 2. Acumox and Tuina Research Section, College of Acumox and Tuina, Shanghai University of Traditional Chinese Medicine,Shanghai, China; 3. Shanghai Research Institute of Acupuncture and Meridians, Shanghai ,China; 4. Acupuncture Department, Shanghai Municipal Hospital of traditional Chinese medicine Affiliated to Shanghai University of TCM, Shanghai, China）   YS and LH contributed equally to this paper. YS and LH are in charge of composition. CZ and SX are in charge of the steering committee. QJ,LH and BL are in charge of coordinating center. LY and SX are in charge of the endpoint adjudication committee. KZ, HJ, and BZ are in charge of the data management team. All authors read and approved the final manuscript. |
|  | 5b | Yin Shou, Email: [33359879@qq.com](mailto:33359879@qq.com) Add: 85 Wujin Road, Hongkou Distrct, Shanghai, China |
|  | 5c | YS and LH contributed equally to this paper. YS and LH are in charge of composition. CZ and SX are in charge of the steering committee. QJ,LH and BL are in charge of coordinating center. LY and SX are in charge of the endpoint adjudication committee. KZ, HJ, and BZ are in charge of the data management team. All authors read and approved the final manuscript. Both YS and BZ ultimate authority over each of these activities. |
|  | 5d | Data monitoring and management will be performed every 3 months by the Clinical Research Center of Shanghai General Hospital Affiliated to Shanghai Jiaotong University. The clinical research monitor will monitor the medical practitioners to ensure all processes are correctly implemented. A data monitoring committee (DMC) has been established independent of the sponsor and no conflict of interest exists. The DMC is responsible for monitoring the progression of the trial and guaranteeing patient safety. Interim analyses and stopping plans for the trial have not been specified, but if the DMC requests interim analyses, these will be supplied. Two assistants will enter all data into an electronic database by double data entry. The statistical manager will be responsible for source data organizing, coding, range checking for data values and converting data to ensure data quality. The database will be locked after all data has been cleaned. If subjects withdraw from the trial, the reasons should be detailed and the rate of withdrawal statistically analyzed. |
| **Introduction** |  |  |
| Background and rationale | 6a | Many previous studies have shown the potential therapeutic effect of acupuncture for allergic rhinitis. Most of these studies were limited by low-grade evidence. Preliminary experiments show that the use of acupuncture at three nasal points plus acupoint application (AAP) achieves a much more persistent effect in the treatment of perennial allergic rhinitis compared with acupuncture therapy alone. Therefore, a multicenter, single-blind, randomized controlled trial has been designed in which acupuncture at non-meridian points and sham AAP will be used as the control group, and the effect of AAP therapy will be evaluated through long-term observation. |
|  | 6b | Based on this preliminary research, a multicenter, randomized controlled trial (RCT) has been designed, in which acupuncture at non-meridian point and sham AAP will be used with a control group, so that the effect of AAP therapy can be evaluated through long-term observation. Similar research techniques have been applied in previous studies on PAR, such as Total Nasal Symptom Score (TNSS) ,Visual Analog Scale (VAS) , and Total Non-Nasal Symptom Score (TNNSS). This study has adopted TNSS, VAS, and TNNSS due to their wide applicability. |
| Objectives | 7 | The study’s hypothesis is that acupuncture at three nasal points plus AAP will achieve equal or better long-term symptom relief of perennial or severe AR and reduce its recurrence. |
| Trial design | 8 | A multicenter, RCT for patients with PAR has been designed, using acupuncture at non-meridian points and sham AAP as a control. |
| **Methods: Participants, interventions, and outcomes** | | |
|  |  |  |
| Study setting | 9 | Patients will be recruited from three hospitals:   1. Shanghai General Hospital Affiliated to Shanghai Jiaotong University 2. Shanghai Traditional Chinese Medicine Hospital 3. Shanghai Research Institute of Acupuncture and Meridians   All three research centers are situated in Shanghai, China. |
| Eligibility criteria | 10 | Inclusion criteria  Participants will be eligible if they meet all of the following criteria: 1) Consistent with moderate of serious severe PAR diagnostic criteria, 2) Consistent with PAR lung qi deficiency and cold syndrome diagnostic criteria, 3) Gender not limited, but aged 18–60 years old, 4) PAR is distinguished from cold or tetanus-related motor rhinitis, and 5) Patient agrees to participate in the study with informed consent.  Exclusion criteria  Patients will be excluded if they have any of the following conditions: 1) Not consistent with PAR diagnostic criteria, 2) Combined with rhinosinustis, sinusitis, deviation of nasal septum, 3) Combined with serious breathing illness, circulation disease, digestion disease, urinary system disease, hematology disease, nervous system disease, endocrine system disease, mental disorder, or malignant tumor, 4) Antibiotics for the treatment of upper respiratory tract infection or paranasal sinusitis have been used in the two weeks prior to the start of the study, 5) Women who are pregnant or lactating, 6) Skin lesions or scars are present on the AAP point, 7) Patient is especially sensitive to drugs or dressings, 8) The TNSS before treatment is below 4, and 9) The presence of alcoholism, which cannot be terminated during the experiment. |
| Interventions | 11a | Treatment group  All licensed acupuncture physicians have completed at least five years of undergraduate education and are registered TCM practitioners. All research assistants and licensed acupuncturists involved in the trial will receive a 2-day training session prior to the start of the study. Both treatments will consist of 12 sessions, each of 30 minutes duration, administered regularly over a 4-week period. The licensed acupuncturists will provide acupuncture at three nasal points and AAP three times a week for 4 weeks.  Participants in the treatment group will receive acupuncture at three nasal acupoints and AAP. The acupuncture needles are disposable and sterile and measure 0.25 × 40 mm (Suzhou Tianxie Acupuncture Instruments Co., Ltd., Suzhou, China). The acupuncture acupoints will be the three nasal points Yintang (EX-HN3), Yingxiang (LI20), and Shangyingxiang (EX-HN8), as well as Feishu (BL13), Dazhu (BL11), Fengmen (BL12), Taiyuan (LU9), Zusanli (ST36), which will be located according to World Health Organization (WHO) International Standard Acupuncture Points. Patients will be in a seated position during treatment sessions. After routine skin sterilization, needles will be inserted using a neutral reinforcing and reduced manipulation technique. Each needle will be rotated until the patient experiences the qi feeling of soreness, heaviness, and the sensation of distension. The complete AAP formula for this study is not publicly available, but the primary herbal ingredients include Rhizoma corydalis, Semen sinapis, Ephedra sinica, Cortex cinnamomi, Euphorbia kansui, Syzygium aromaticum, Asarum sieboldi Mig. The herbs are processed into powder, proportionally mixed at a 2:2:1:1:1:1:1 ratio, and blended in fresh ginger juice to create an AAP ointment, which is then mechanically poured into tubes. The resulting AAP ointment is stored in a refrigerator at 4˚C. The AAP and matching placebo are both manufactured by the Pharmaceutical Preparation Department of the First People's Hospital Affiliated to Shanghai Jiaotong University, and both meet regulatory guidance requirements issued by the China Food and Drug Administration. For each acupoint, approximately three grams of ointment is squeezed by assistants onto a 6 cm diameter circular fabric. For each participant, the following six acupoints will be used: Dazhui (GV14), Feishu (BL13), Dingchuan (EX-B), Tiantu (CV22), located according to WHO International Standard Acupuncture Points. The procedure for conducting therapy has been standardized in each center by advance training (see Table [2](https://www.ncbi.nlm.nih.gov/pmc/articles/PMC4929718/table/Tab2/)).  Control group  Participants in the control group will receive shallow needling at sham Yintang (EX-HN3), sham Yingxiang (LI20), sham Shangyingxiang (EX-HN8), sham Feishu (BL13), sham Dazhu (BL11), and sham Taiyuan (LU9). Each of these is a non-acupoint located at a different physical location to the actual acupoints. The placebo ointment is composed of buckwheat powder and coke, resulting in an ointment similar in appearance to the AAP ointment. |
|  | 11b | The intervention is to be terminated in the case of severe adverse events, withdrawal of participants by their own choice and unpermitted medication use. |
|  | 11c | Use placebo induction to exclude subjects with low compliance |
|  | 11d | Concomitant care and intervention  In both groups, participants with severe symptoms will be allowed to use rescue medicine with documentation of the medication. The type of medicine, dosage, and usage will be recorded on diary cards for analysis. For more complicated chronic diseases, patients must continue to take their routine medication and to receive the necessary therapies. In the patient’s case report, research staff will record the names of these diseases and the names of medications and therapies used. |
| Outcomes | 12 | Outcome measures  Baseline information  Demographic information will be collected using a custom-made, standardized survey form that includes: center location, name, age, gender, address, telephone number, and employment. Medical information will be collected using a custom-made form that collects clinical characteristics information, including diagnosis, nasal mucosal examination result, allergen examination result, typical symptoms, occurrence time of symptoms, accessory examination of nasal mucosa and nasal sinus, relevant diseases (allergic asthma, allergic conjunctivitis), and medication history.  Primary outcome measure  Average change in TNSS will be blind measured by comparing the baseline score of each group with the score at the end of the 4-week treatment. TNSS is determined by severity of rhinorrhea, nasal itching, nasal obstruction, and sneezing.  Secondary outcome measures  Average change in TNSS will be measured by comparing the baseline score of each group with the score after 2 weeks of treatment, and 1, 3, and 6 months after treatment. VAS and TNNSS will be used to observe supplementary symptoms and degree of symptoms. These will also be evaluated at 2 and 4 weeks, and 1, 3 and 6 months after treatment. Patients’ peripheral blood IL-4, IL-5, IL-6, IL-8, and IL-10 will be measured by Luminex to observe allergic reactions.  All outcome readings will be scored on quantitative scales, and summarized as mean values and standard deviation. |
| Participant timeline | 13 | For an overview of the recruitment timeline, interventions and all time points of subject evaluation, see Fig. 2. |
| Sample size | 14 | Sample size calculation  The trial will test two groups in parallel. The sample size calculation was performed using SAS 9.3 software (SAS Institute Inc., Cary, NC, USA) in the Clinical Evaluation Center of First People's Hospital Affiliated to Shanghai Jiaotong University. The mean change in TNSS before and after treatment was used as the indicator of efficacy evaluation in the calculation of the sample size. Results from other research has shown a mean TNSS change of 2.53 ± 4.74 after acupuncture treatment (25) and 2.75 ± 1.06 after AAP (36). Based on these findings, a test was performed to calculate the appropriate trial sample size with 80% power, alpha of 0.05, and an acceptable delta of 0.2. The results show that a clinically important difference can be detected by a sample size with at least 49 in each group. The number was then increased to 60 per group (total of 120) to allow for a predicted 20% dropout rate. |
| Recruitment | 15 | In order to maximize the retention of subjects in the study, the working attitude of all staff members is very important. Ask subjects about their difficulties and offer appropriate arrangements or adjustments to facilitate their continued participation in the study. Be kind to our subjects, always listen patiently and initiate conversations with them. During follow-up, the investigator and the clinical research coordinator (CRC) must pay attention to the subjects' psychology. Some problems can be solved if problems are detected early. For example, if the subject has difficulty in getting to the hospital, the round-trip fare can be reimbursed or a special bus can be arranged, especially for the elderly subjects. One day in advance of each follow-up visit, and 2 to 3 days before each follow-up visit, the study nurse should call to remind the subjects of the time of the last follow-up visit. For patients who have difficulty following up at the investigator's regular clinic, additional follow-up times can be arranged, such as evenings or weekends. |
| Methods: Assignment of interventions (for controlled trials) | | |
| Allocation: |  |  |
| Sequence generation | 16a | Participants will be randomly assigned in a 1:1 ratio to either the treatment group or the control group. The randomization sequence was generated using blocked randomization with a table of randomization. The table contains, in random order, all possible combinations of a small series of figures, and assumes equal probability of patients being randomly assigned to treatment group or control group. The order of the interventions assigned to each block is randomized. The process is repeated for consecutive blocks until all participants are randomized. |
| Allocation concealment mechanism | 16b | While receiving the first treatment, participants will be given sequential treatment cards from independent researchers to ensure adequate concealment. |
| Implementation | 16c | Participants are allocated a numerical code for data entry into SPSS. Paper copies of consent forms and completed questionnaires will be stored in a locked filing cabinet at the hospital. Electronic data will be stored on a password protected computer hard drive that can only be accessed by the research team. |
| Blinding (masking) | 17a | This will be a single-blind trial. All participants will be treated separately to prevent communication. Except for acupuncturists, all relevant parties will be blind to the intervention groups. The implementation of treatments is performed by two acupuncturists using patches prepared by operational assistants. Due to the nature of acupuncture and acupoint application, it is difficult to fully ensure blinding among participants allocated to either treatment or control group. Participants will be required to wait for 120 minutes in a room, after which their treatment patches will be removed by research nurses. In addition, acupuncturists, operational assistants, and research nurses are instructed to not communicate to participants anything that might alert them to which group they have been allocated to. Participants will be informed that they have an equal chance of allocation to the treatment group or control group before research participation. The participants will be blind to which group they belong to, as needle penetration is achieved in each case. |
|  | 17b | In addition, outcome evaluators and statistical analysts will be blind to the groupings and will not be involved in any part of the treatments during trial, to ensure no bias in the results. |
| Methods: Data collection, management, and analysis | | |
| Data collection methods | 18a | Data collection and management  Study staff will be responsible for the collection of baseline characteristic data and medical results during the screening period. For reasons of convenience, all subjects’ scores, observation times, AE records, and safety assessments will be consolidated into a single CRF. CRFs must be filled out immediately and accurately. Participants are required to record, in their daily diary, any other medications they take during this study period. Outcome evaluators will examine the outcomes at baseline, 2 weeks (within treatment), 4 weeks (end of treatment), 1 month (within follow-up), 3 months (within follow-up), and 6 months (end of follow-up). Data on nasal symptoms and non-nasal symptoms will be collected.  Data monitoring and management will be performed every 3 months by the Clinical Research Center of First People's Hospital Affiliated to Shanghai Jiaotong University. The clinical research monitor will monitor the medical practitioners to ensure all processes are correctly implemented. A data monitoring committee (DMC) has been established independent of the sponsor and no conflict of interest exists. The DMC is responsible for monitoring the progression of the trial and guaranteeing patient safety. Interim analyses and stopping plans for the trial have not been specified, but if the DMC requests interim analyses, these will be supplied. Two assistants will enter all data into an electronic database by double data entry. The statistical manager will be responsible for source data organizing, coding, range checking for data values and converting data to ensure data quality. The database will be locked after all data has been cleaned. If subjects withdraw from the trial, the reasons should be detailed and the rate of withdrawal statistically analyzed. |
|  | 18b | To improve adherence to the intervention, free treatment and free blood tests will be provided to the treatment group for 4 weeks. To ensure that treatment and follow-up run to schedule, participants will be assured of and given monetary compensation at the end of the follow-up period. |
| Data management | 19 | To ensure the authenticity of the data, a special research team from the Clinical Research Center of Shanghai General Hospital Affiliated to Shanghai Jiaotong University, independent of the investigators and the sponsors, will externally monitor the study in the three hospitals every 3 months. An advisory board will follow the trial and provide advice when necessary. |
| Statistical methods | 20a | Analysis procedures  Statistical analysis will be performed using SPSS 16.0 software (SPSS Inc., Chicago, IL, USA) in the Clinical Evaluation Center of Shanghai General Hospital Affiliated to Shanghai Jiaotong University. |
|  | 20b | Baseline information  Baseline adjusted analyses will be provided for center and severity variables, and the baseline value of the corresponding outcomes will be assessed. Descriptive statistics will be used to compare baseline measures with patients’ characteristics. If an imbalance occurs in baseline characteristics between the two groups, ANCOVA will be applied.  Efficacy analysis  Efficacy data analyses will be conducted on an intention-to-treat population. All subjects initially included in one of the two groups will be considered in the statistical analysis. Analysis of efficacy will be carried out per-protocol and will include all subjects who complete the entire research process. Descriptive statistics will be used to compare serum indicators between the two groups. Regarding the primary and secondary outcome measures, a two-sample t test or Wilcoxon rank sum test will be used to compare differences between the two groups from baseline to the end of treatment (p < .05 will be considered statistically significant). The mean and standard deviations of these parameters will be reported. If the data come from repeated measurements, the RMANOVA method will be used after meeting spherical symmetry requirements. SPSS 16.0 software will be used for all statistical calculations.  Safety analysis  According to the definition of AEs, AEs will be recorded, along with their severity level, causes, and explanations. The number of AEs, and the rate of AE, will be described statistically. If AEs need to be compared between groups, theχ2 test or Fisher’s exact test will be used. |
|  | 20c | Missing data analysis  All data used in the main statistical analysis should be collected by the fourth week of the treatment and by the half-year follow-up. To avoid missing data, participants who complete the trial and provide completed data will be financially compensated. The investigators have a wealth of experience from previous trials in managing patients and collecting data. Patient contact information will be recorded and researchers will keep in touch with patients through a variety of means during the treatment and follow-up periods.  If data are not obtained, the time and reason for the missing data will be recorded and the assumed missing data mechanism will be analyzed. For these missing data, a multiple imputation adjustment approach will be used. After the main analysis, a sensitivity analysis will be performed for the various datasets to enable an assessment of the impact of missing data on the results.  A fully specified statistical analysis plan will be written independently. |
| Methods: Monitoring | | |
| Data monitoring | 21a | Data monitoring and management will be performed every 3 months by the Clinical Research Center of First People's Hospital Affiliated to Shanghai Jiaotong University. The clinical research monitor will monitor the medical practitioners to ensure all processes are correctly implemented. A data monitoring committee (DMC) has been established independent of the sponsor and no conflict of interest exists. The DMC is responsible for monitoring the progression of the trial and guaranteeing patient safety. Interim analyses and stopping plans for the trial have not been specified, but if the DMC requests interim analyses, these will be supplied. Two assistants will enter all data into an electronic database by double data entry. The statistical manager will be responsible for source data organizing, coding, range checking for data values and converting data to ensure data quality. The database will be locked after all data has been cleaned. If subjects withdraw from the trial, the reasons should be detailed and the rate of withdrawal statistically analyzed. |
|  | 21b | Should the participant become distressed, the intervening practitioner will debrief the participant and sign post him or her to appropriate support services. Any adverse events that are observed will be reported to the steering committee, who will consider the criteria for discontinuing or modifying the allocated intervention. Serious breaches of protocol will be reported to the trial sponsor. The independent steering committee will also make the final decision to terminate the trial prematurely, should this appear to be the appropriate course of action. |
| Harms | 22 | Adverse events (AEs) are defined as at least four subjects suffering from the same symptom which is any undesirable experience occurring to participants during the trial period. This may or may not be associated with the intervention. Participants are instructed to report any AE to the research team at any time. All details of AEs, including time of occurrence, description of symptoms, duration of symptoms, severity, management measures, and causality to the intervention, are recorded on case report forms (CRFs). Common AEs related to acupuncture include local skin pain, itching, ulcers, needle left in participant, nausea during acupuncture, fainting during acupuncture, severe sharp pain, sharp pain lasting more than 30 minutes, hematoma around the site of needling, bleeding, numbness, infection around the site of needling, sleeplessness after acupuncture, dizziness after acupuncture, and so on. Common AEs related to AAP include local itching, redness, and blisters. The causality between AEs and intervention is assessed according to the WHO Uppsala Monitoring Center System for Standardized Case Causality Assessment. If AEs occur, staff will select an appropriate treatment method until the condition has stabilized. After the subject’s condition returns to normal, staff will decide whether further observation is required. Severe AEs must be reported to the safety monitoring board within 24 hours of their occurrence. |
| Auditing | 23 | Shanghai Jiaotong University will act as sponsor for the research and will undertake regular audit. The Programme Management Group will be responsible for ensuring the appropriate and timely implementation of the trial. |
| **Ethics and dissemination** | | |
| Research ethics approval | 24 | The study has been approved by the Ethics Committee of the Institute of Shanghai General Hospital Affiliated to Shanghai Jiaotong University ([2017]31), Shanghai Municipal Hospital of Traditional Chinese Medicine (2017SHL-KY-06), and the Ethics Committee of Shanghai Research Institute of Acupuncture and Meridians (2017-037-01). Having obtaining ethics committee approval, the trial was registered on an authoritative registration platform for clinical trials (Acupuncture and Moxibustion Clinical Trial Registry, [AMCTR-ICR-](https://clinicaltrials.gov/ct2/show/NCT02339714)18000179). |
| Protocol amendments | 25 | Any future minor and substantial protocol amendments will be submitted to the above ethics committee for ethical consideration and future changes to protocol will be included in publications. |
| Consent or assent | 26a | If the patients decide to participate, they will be asked to provide written consent, which will be recorded on a consent form (please see consent form in the Additional file 2). |
|  | 26b | Additional consent will also be sought for qualitative interviews which will be conducted within the process evaluation. The consent form will include a unique identifier which enables the consent to be linked to the anonymised personal data gathered within the baseline questionnaire. |
| Confidentiality | 27 | All identifiable data collected will be separated from personal data to maintain confidentiality and a unique reference number allocated to enable data linkage across sources and time periods. All data will be stored on a secure server, with identifiable data being password protected and stored. |
| Declaration of interests | 28 | The authors declare no competing interests. |
| Access to data | 29 | The datasets generated during and/or analyzed during the current study are not publicly available because data sharing would require institutional approval but are available from the corresponding author on reasonable request. |
| Ancillary and post-trial care | 30 | Researchers use treatments under clinical trial protocols, which require financial compensation if a subject's health is harmed during a trial. If there is a factual causal relationship between the clinical trial therapy and the health of the subject, the subject should be compensated. |
| Dissemination policy | 31a | Publication and dissemination  Following the completion of data analysis, Chinese and English language dissemination is planned. Regardless of the findings, the trial results with be disseminated via conferences or publications.  All of the staff who participated in the organization, implementation, data management, and data statistical analysis will be affirmed in the authorship, and there is no intention to use professional writing services.  There is no plan to permit public access to the full protocol, participant dataset, or statistical code. However, if necessary, individuals can gain access to the full protocol through the Ethics Committee of the Institute of Shanghai General Hospital Affiliated to Shanghai Jiaotong University.  This protocol was written following the SPIRIT checklist (see Additional file [1](https://www.ncbi.nlm.nih.gov/pmc/articles/PMC4929718/#MOESM1)). The future report will follow the CONSORT guidelines, Revised Standards for Reporting Interventions in Clinical Trials of Acupuncture, and the extension of CONSORT for reporting single-blind randomized trials. |
|  | 31b | Authorship of future published outputs will include authors who have made a substantial contribution to the conception or design of the work; or the acquisition, analysis or interpretation of data for the work, including drafting and authorising of manuscripts. Trial findings will also be communicated to trial participants within a lay summary. |
|  | 31c | Data sharing will be open to the public within 12 months after the completion of the trail. |
| **Appendices** |  |  |
| Informed consent materials | 32 | Participants will be required to sign the informed consent forms before the trial. Research assistants will be in charge of the storage of all informed consent forms. |
| Biological specimens | 33 | Storage of biological specimens for genetic or molecular analysis in the current trial and for future use will be kept under the guidelines for laboratory management of biological sample analysis in clinical trials. |
